# Generalist genes and the Internet generation: etiology of learning abilities by web testing at age 10

**DOI:** 10.1111/j.1601-183X.2007.00370.x

**Published:** 2008-06

**Authors:** O S P Davis, Y Kovas, N Harlaar, P Busfield, A McMillan, J Frances, S A Petrill, P S Dale, R Plomin

**Affiliations:** † Social, Genetic and Developmental Psychiatry Centre, Institute of Psychiatry King's College London, London, United Kingdom; ‡ Department of Human Development and Family Science, Ohio State University Columbus, OH; § Department of Speech and Hearing Sciences, University of New Mexico Albuquerque, NM, USA

**Keywords:** General cognitive ability, generalist genes, genetic correlation, Internet, mathematics, reading, twins

## Abstract

A key translational issue for neuroscience is to understand how genes affect individual differences in brain function. Although it is reasonable to suppose that genetic effects on specific learning abilities, such as reading and mathematics, as well as general cognitive ability (*g*), will overlap very little, the counterintuitive finding emerging from multivariate genetic studies is that the same genes affect these diverse learning abilities: a *Generalist Genes* hypothesis. To conclusively test this hypothesis, we exploited the widespread access to inexpensive and fast Internet connections in the UK to assess 2541 pairs of 10-year-old twins for reading, mathematics and *g*, using a web-based test battery. Heritabilities were 0.38 for reading, 0.49 for mathematics and 0.44 for *g*. Multivariate genetic analysis showed substantial genetic correlations between learning abilities: 0.57 between reading and mathematics, 0.61 between reading and *g*, and 0.75 between mathematics and *g*, providing strong support for the Generalist Genes hypothesis. If genetic effects on cognition are so general, the effects of these genes on the brain are also likely to be general. In this way, generalist genes may prove invaluable in integrating top-down and bottom-up approaches to the systems biology of the brain.

Naturally occurring genetic variation is a Rosetta Stone for translating causal effects from genes to brain to cognition, especially once specific genes are identified (Plomin *et al.* in press). Although learning abilities and disabilities are highly heritable, specific genes responsible for their heritability have not yet been identified despite several promising candidate genes for reading disability (Fisher & Francks 2006). Nonetheless, more can be gleaned from quantitative genetic research, such as twin studies that compare identical and fraternal twins, than the mere fact that they are heritable. A major advance in quantitative genetics is multivariate genetic analysis, which investigates not only the variance of traits considered one at a time but also the covariance among traits. In this way, it indicates the extent to which the same or different genes affect several traits (Neale *et al.* 2006), using a statistic known as a *genetic correlation*(Plomin *et al.* in press). These findings can constrain explanations of brain processes that underlie the traits. For example, it is reasonable to suppose that genetic effects will be specific to the substantially different cognitive processes involved in reading and mathematics. Such genetic specificity would indicate the need to identify the genetically driven differences in brain processes that underlie these cognitive differences.

However, a very different result is emerging from multivariate genetic research on learning abilities and disabilities. Most genetic effects appear to be general in that the same genes affect different learning abilities and disabilities. A review of multivariate genetic research on learning abilities found that genetic correlations varied from 0.67 to 1.0 between reading and language (five studies), 0.47 to 0.98 between reading and mathematics (three studies) and 0.59 to 0.98 between language and mathematics (two studies) (Plomin & Kovas 2005). The average genetic correlation was about 0.70. Moreover, the general effects of genes appear to extend beyond specific learning abilities such as reading and mathematics to other cognitive abilities such as verbal abilities (e.g. vocabulary and word fluency) and non-verbal abilities (e.g. spatial and memory). The average genetic correlation between specific learning abilities and general cognitive ability (*g*), which encompasses these verbal and non-verbal cognitive abilities, is about 0.60 (Plomin & Kovas 2005). These findings have led to a *Generalist Genes* hypothesis (Plomin & Kovas 2005), which has far-reaching implications for cognitive neuroscience (Kovas & Plomin 2006). However, the Generalist Genes hypothesis has yet to be tested by direct cognitive test measures in a sample large enough to conclusively establish the magnitude of the genetic correlations between learning abilities. To address this problem, we developed an online test battery that includes measures of reading, mathematics and *g* and used it to assess a UK-representative population sample of 2541 pairs of 10-year-old twins: by far the largest twin sample with cognitive test data. The purpose of the present study was to exploit the potential of web-based administration to provide a powerful test of the Generalist Genes hypothesis.

## Methods

### Participants

The Twins Early Development Study (TEDS) recruited families of twins born in England and Wales in 1994, 1995 and 1996: three annual cohorts (Oliver & Plomin 2007; Trouton *et al.* 2002). The present paper describes results at 10 years, where a two-cohort subsample of TEDS (twins born in 1994 and 1995) was tested. Despite inevitable attrition (Oliver & Plomin 2007), the sample remains representative of the UK population (ascertained by comparison with census data from the Office of National Statistics). Notably, because of the widespread availability of fast Internet access, we found no evidence of sampling bias in favor of higher socioeconomic status families. Informed consent was obtained by post or online consent forms, and a test administrator was then assigned who telephoned the family, sorted out any problems with the Internet or testing and generally assisted and encouraged the participating family. Ethical approval for TEDS has been provided by the Institute of Psychiatry Ethics Committee, reference number 05/Q0706/228.

We excluded from the analyses children with severe current medical problems and children who had suffered severe problems at birth or whose mothers had suffered severe problems during pregnancy from the analyses. We also excluded twins whose zygosity was unknown or uncertain or whose first language was other than English. Finally, we included only twins whose parents reported their ethnicity as ‘white’, which was 93% of this UK sample. The present analyses are based on 2541 twin pairs [919 monozygotic (MZ) pairs, 817 same-sex dizygotic (DZ) and 805 opposite-sex DZ].

### Internet testing

Widespread access to inexpensive and fast Internet connections in the UK has made online testing an attractive possibility for collecting data on the substantial samples necessary for genetic research, especially for multivariate genetic research. The advantages and potential pitfalls of data collection over the Internet have been reviewed in detail elsewhere (Birnbaum 2004). For older children, most of whom are competent computer users, it is an interactive and enjoyable medium. Through adaptive branching, it allows the use of hundreds of items to test the full range of ability, while requiring individual children to complete only a relatively small number of items to ascertain their level of performance. In tests where it is appropriate, streaming voiceovers can minimize the necessary reading. In addition, the tests can be completed over a period of several weeks, allowing children to pace the activities themselves, although they are not allowed to return to items previously administered. Finally, it is possible to intersperse the activities with games. All of these factors help maintain children's engagement with the tests.

### Measures

Reading was assessed by an adaptation of the Peabody Individual Achievement Test (PIAT-Revised; Markwardt 1997) Reading Comprehension Scale, and mathematics by three subtests based on the nferNelson Math 5-14 Series (nferNelson 2001): Understanding Number, Non-Numerical Processes and Computation and Knowledge. *g* was indexed by two verbal tests – WISC-III-PI multiple-choice Vocabulary and Information (Wechsler 1992) – and two non-verbal tests – WISC-III-UK Picture Completion (Wechsler 1992) and Raven's Standard Progressive Matrices (Raven *et al.* 1996). We created a *g* score with equal weights for the four tests by summing their standardized scores. This unit-weighted score correlated 0.99 with the corresponding factor score. Further information about the measures is available elsewhere (Oliver & Plomin 2007). We have shown that the web-based tests are reliable, stable and valid (Haworth *et al.* 2007). As an index of reliability, Cronbach's alpha was 0.95 for PIAT subtests, 0.78–0.93 for the math subtests and 0.74–0.91 for the *g* subtests (*n* = 2569–2924). In terms of stability and validity, scores on the online version of our reading and mathematics tests correlate highly with traditional in-person versions administered in person 1–3 months later: *r* = 0.80 for PIAT and 0.92 for mathematics (*n* = 30). Download time, a proxy for computer performance, accounted for less than 2% of the variance in the PIAT and less than 0.5% of the variance in the other tests.

### Statistical analyses

According to the quantitative genetic model (Plomin *et al.* in press; Rijsdijk & Sham 2002), same-sex twins reared together resemble each other because of the additive effects of shared genes (A) or shared (common) environmental factors (C). For identical or MZ twins, the correlation between their genes is 1.00, whereas for non-identical or DZ twins, the correlation is 0.50 because DZ twins on average share half of their segregating alleles. The correlation between twins for shared environment is, by definition, 1.00 for both MZ and DZ twins growing up in the same family, while non-shared environmental influences (E) are uncorrelated and contribute to differences between twins. For the twin analyses, standardized residuals correcting for age and sex were used because the age of twins is perfectly correlated across pairs, which means that, unless corrected, variation within each age group at the time of testing would contribute to the correlation between twins and be misrepresented as shared environmental influence (Eaves *et al.* 1989). The same applies to the sex of the twins because MZ twins are always of the same sex. Likewise, download time, a proxy for computer performance, was also regressed out of the twins’ test scores. The assumptions of the classical twin model, and their validity, have been discussed in detail elsewhere (Boomsma *et al.* 2002; Martin *et al.* 1997; Rijsdijk & Sham 2002).

As well as examining twin correlations, we used standard *ACE* model-fitting analysis in Mx1.7.01 (Neale *et al.* 2006) where ACE stands for additive genetic influences (A), shared or common environmental influences (C) and non-shared environmental influences (E), as above. Model-fitting analysis specifies a correlational structure (a model) using matrix algebra. This model is a hypothesis about the structure of the dataset and is derived from what we know about how MZ and DZ twins are related to each other (see above). By fitting the model to the data using an iteration process, we can derive its ‘goodness of fit’ and parameter estimates for the contributions of A, C and E. Before embarking on our multivariate analysis, we initially examined sex differences in the genetic and environmental parameter estimates by comparing the fit of three full univariate ACE models (one each for reading, mathematics and *g*) with that of various nested models, dividing the twin pairs into five groups: MZ male, MZ female, DZ male, DZ female and DZ opposite-sex pairs. These sex-limitation models (Eley 2005) allowed us to estimate qualitative and quantitative etiological differences between the sexes (Galsworthy *et al.* 2000).

**Figure 1:**
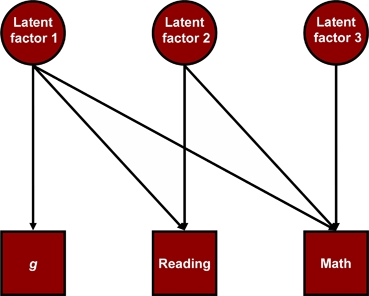
**Path diagram for the full phenotypic Cholesky decomposition model.**The Cholesky partitions variance into a universal factor influencing all three traits (latent factor 1), a factor influencing reading and mathematics (latent factor 2) and a factor influencing mathematics alone (latent factor 3).

**Figure 2:**
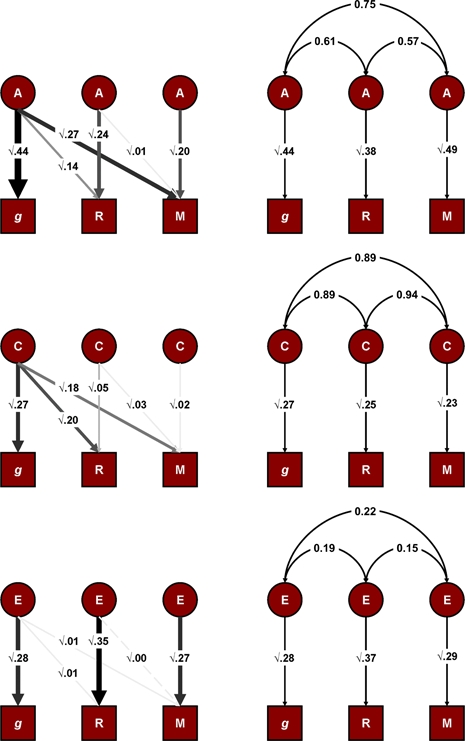
**Multivariate analysis.**The top panel gives the estimates for the additive genetic (A) component of the variance, with the Cholesky solution on the left and the correlated factors solution on the right. The middle panel does the same for the shared environment (C) and the bottom panel gives the estimates for non-shared environmental effects (E). In the Cholesky diagrams, line weights and intensities represent strength of association. Dotted paths can be dropped individually without a significant (*P* > 0.05) decrement in model fit. In the correlated factors diagrams, the curved arrows represent correlations between the latent factors. R, reading; M, mathematics.

Finally, we used multivariate genetic model fitting to investigate the genetic and environmental etiology of the covariation between learning abilities. Figure 1 shows the phenotypic Cholesky decomposition, which partitions variance into a universal factor influencing all three traits, a factor influencing reading and mathematics independent of *g* and a factor influencing mathematics independent of reading and *g*. As shown on the left of Fig. 2, the genetic Cholesky decomposition partitions this variance further into genetic, shared environmental and non-shared environmental components. As in the phenotypic Cholesky, variance attributable to genetic influences is divided into a universal genetic factor influencing *g*, reading and mathematics (A_1_); a genetic factor specific to reading and mathematics (A_2_) and a genetic factor unique to mathematics (A_3_). The shared environmental influences (C_1_, C_2_, C_3_) and non-shared environmental influences (E_1_, E_2_, E_3_) are partitioned in the same way.

The correlated factors model (on the right) can be derived from the Cholesky model. It specifies three latent genetic factors, one for each trait, and calculates the correlation between them. Once again, the same is true for the shared and non-shared environments. These statistics, the genetic, shared environmental and non-shared environmental correlations, are unique to multivariate analysis. Along with bivariate heritability and environmentality (the proportion of the phenotypic correlation accounted for by genes or the environment), they give us an essential insight into how genetic factors and environments are shared in the etiology of learning abilities. It is important to note that the genetic and environmental correlations are independent of the heritability or environmentality of the traits. For example, two traits with very little heritability can nevertheless be highly correlated genetically (i.e. share the same genetic influence), and two highly heritable traits can be genetically uncorrelated (independent genetic influences).

In Fig. 2, the squared path coefficients influencing each measured variable in the correlated factors model can be derived from the corresponding squared paths in the Cholesky model. Finally, individual Cholesky pathways were dropped one at a time, and the fit was compared with the full model. This tests the statistical significance of the influence of each latent factor on *g*, reading and mathematics and indicates the most parsimonious model.

## Results

### Phenotypic analyses

As shown in Table 1, the measures exhibited small mean differences for sex (boys higher for mathematics and *g*) and zygosity (DZs higher for reading and *g*), although the differences are statistically significant, given the large sample sizes. Altogether, sex and zygosity accounted for less than 1% of the variance in all measures.

**Table 1:** Measure means (M) and standard deviations (SDs) by sex and zygosity

Measure	MZM		DZM		DZOM		MZF		DZF		DZOF		anova*P* values			*R*^2^	*n*
	M	SD	M	SD	M	SD	M	SD	M	SD	M	SD	Sex	Zygosity	Sex × Zygosity		
Mathematics	0.04	1.02	0.15	1.01	0.03	1.01	−0.11	0.96	−0.05	1.05	0.00	0.94	0.00**	0.11	0.49	0.01	4922
Reading	−0.06	1.06	0.04	1.04	0.04	1.02	−0.06	0.96	0.02	1.00	0.05	0.93	0.92	0.00**	0.90	0.00	5351
*g*	0.06	0.97	0.14	0.96	0.05	1.02	−0.11	1.03	−0.08	1.03	0.00	0.95	0.00**	0.04*	0.58	0.01	4684

DZF, DZ females; DZM, DZ males; DZOF, females in DZ opposite-sex pairs; DZOM, males in DZ opposite-sex pairs; MZF, MZ females; MZM, MZ males.

* *P* values significant at the <0.05 level.

** *P* values significant at the <0.01 level.

Phenotypic correlations for the age-, sex- and download-time-regressed indicators were as follows (using one member of each twin pair): mathematics–reading, *r* = 0.51, *n* = 2457, *P* = 0.000; mathematics–*g*, *r* = 0.63, *n* = 2342, *P* = 0.000; reading–*g*, *r* = 0.54, *n* = 2333, *P* = 0.000.

### Univariate genetic analyses

Intraclass correlations (Shrout & Fleiss 1979; twin similarity coefficients) are shown in Table 2 for the total group of MZ, DZ same-sex and DZ opposite-sex twins, as well as for the male and female subgroups among the same-sex twin pairs. Correlations between MZ twins were consistently higher than those between DZ twins, suggesting a genetic contribution to reading, mathematics and *g*. As a first estimate, doubling the difference between the MZ and same-sex DZ correlations yields moderate heritability estimates of 40% for mathematics, 40% for reading and 36% for *g*. Shared environmental influences are also moderate, estimated as the extent to which MZ resemblance exceeds heritability: 31% for mathematics, 24% for reading and 35% for *g*. The remainder of the variance is attributed to non-shared environmental influences (plus error of measurement): 29% for mathematics, 36% for reading and 29% for *g*.

**Table 2:** Twin similarity coefficients (intraclass correlations) for mathematics, reading and *g*

Measure	MZ	DZS	DZO	DZall	MZM	MZF	DZM	DZF
Mathematics	0.71 (0.68–0.75), (*n* = 863)	0.51 (0.45–0.56), (*n* = 762)	0.43 (0.37–0.49), (*n* = 751)	0.47 (0.43–0.51), (*n* = 1513)	0.69 (0.63–0.74), (*n* = 356)	0.73 (0.68–0.77), (*n* = 507)	0.47 (0.38–0.55), (*n* = 343)	0.54 (0.47–0.60), (*n* = 419)
Reading	0.64 (0.60–0.67), (*n* = 919)	0.44 (0.39–0.50), (*n* = 817)	0.42 (0.36–0.47), (*n* = 805)	0.43 (0.39–0.47), (*n* = 1622)	0.64 (0.58–0.70), (*n* = 383)	0.63 (0.58–0.68), (*n* = 536)	0.43 (0.34–0.51), (*n* = 373)	0.46 (0.38–0.53), (*n* = 444)
*g*	0.71 (0.67–0.74), (*n* = 833)	0.53 (0.47–0.58), (*n* = 728)	0.44 (0.38–0.50), (*n* = 709)	0.48 (0.44–0.52), (*n* = 1437)	0.71 (0.65–0.76), (*n* = 342)	0.71 (0.66–0.75), (*n* = 491)	0.50 (0.42–0.58), (*n* = 328)	0.54 (0.47–0.61), (*n* = 400)

All similarity coefficients are based on age-, sex- and download-time-corrected scores.

95% confidence intervals in parentheses.

Dzall, all DZ pairs; DZO, opposite-sex DZ pairs; DZS, same-sex DZ pairs; MZ, MZ pairs; MZF, MZ female pairs; MZM, MZ male pairs; *n*, number of twin pairs.

As shown in Table 3, ACE model-fitting results are consistent with estimates based on the twin correlations in Table 2. For mathematics, reading and *g*, genetic influence is moderate (49%, 38% and 44%, respectively, for the best-fitting model). Shared and non-shared environmental influences are more modest.

**Table 3:** Parameter estimates for mathematics, reading and *g*

Measure	A	C	E
Mathematics	0.49 (0.40–0.57)	0.23 (0.15–0.30)	0.29 (0.26–0.31)
Reading	0.38 (0.29–0.48)	0.25 (0.17–0.33)	0.37 (0.33–0.40)
*g*	0.44 (0.36–0.53)	0.27 (0.19–0.35)	0.28 (0.26–0.31)

These estimates are based on the best-fitting submodel of the full sex-limitation model, the null model, indicating no quantitative or qualitative differences in etiology between males and females.

95% confidence intervals in parentheses.

A, additive genetic influence; C, shared environmental influence; E, non-shared environmental influence.

Moreover, across zygosity, correlations within male and female pairs (Table 2) were similar, suggesting similar ACE estimates for boys and girls. In addition, correlations between same-sex DZ twins were similar to those between opposite-sex DZ twins, suggesting no qualitative sex differences. Sex-limitation model fitting (Eley 2005) confirmed these expectations, yielding no significant sex differences in ACE parameter estimates or in comparisons between same-sex and opposite-sex DZ twins. The best-fitting model from the sex-limitation analyses was the null model, which includes no quantitative or qualitative differences in etiology between males and females: likelihood ratio χ^2^ test with Δdf compared with full sex-limitation model for mathematics, reading and *g*, respectively: 4.17, 3, *P* = 0.24; 0.18, 3, *P* = 0.98; 3.79, 3, *P* = 0.29. For this reason, and to maximize power, our multivariate genetic analyses combined sexes.

### Multivariate genetic analysis

Cross-trait twin correlations (e.g. twin 1 reading versus twin 2 mathematics) are the essence of multivariate genetic analysis. Table 4 shows that cross-trait twin correlations are consistently greater for MZ than for DZ twins; the MZ cross-trait twin correlations are nearly as great as the phenotypic correlations for the same individual, shown in the third column of Table 4. Doubling the difference between the MZ and DZ cross-trait correlations estimates the genetic contribution to the phenotypic correlations (0.18, 0.32 and 0.32, respectively, for the three rows of Table 4), and dividing these estimates by the phenotypic correlations for the same individual (third column of Table 4) indicates the proportional contribution of genetic influences to the phenotypic correlation: the bivariate heritability (35%, 51% and 59%). As indicated in the *Methods*, the genetic correlation, unlike bivariate heritability, is independent of heritability. The genetic correlation can be estimated by dividing the genetic contribution to the phenotypic correlation by the product of the square roots of the heritabilities of the two traits (Plomin & DeFries 1979). These rough estimates of genetic correlations are substantial: 0.40 for reading and mathematics, 0.73 for reading and *g*, and 0.68 for mathematics and *g*.

**Table 4:** Cross-trait twin similarity coefficients (ICC1.1) for reading, mathematics and *g*

Measures	MZ	DZall	Same individual
Twin 1 reading–twin 2 mathematics	0.46 (0.41–0.51), (*n* = 875)	0.37 (0.33–0.41), (*n* = 1548)	0.51 (0.48–0.54), (*n* = 2457)
Twin 1 reading–twin 2 *g*	0.52 (0.47–0.56), (*n* = 840)	0.36 (0.32–0.41), (*n* = 1462)	0.54 (0.51–0.56), (*n* = 2333)
Twin 1 mathematics–twin 2 *g*	0.56 (0.51–0.61), (*n* = 838)	0.40 (0.36–0.44), (*n* = 1457)	0.63 (0.60–0.65), (*n* = 2342)

All similarity coefficients are based on age-, sex- and download-time-corrected scores. Correlations for same individual are based on one random twin from each pair. Reversing the ordering of the pairs (e.g. twin 2 reading–twin 1 mathematics) produces the same results.

95% confidence intervals in parentheses.

Dzall, all DZ pairs; MZ, MZ pairs; *n*, number of twin pairs.

The results of multivariate model-fitting analyses echo this simple analysis based on the cross-trait twin correlations. The Cholesky decomposition model is shown in Fig. 1. Tables 5 and 6 show parameter estimates and confidence intervals, with the results summarized visually in Fig. 2. As shown in Table 5 and Fig. 2 (curved arrows, top right), genetic correlations are substantial (0.57 for reading and mathematics, 0.61 for reading and *g*, and 0.75 for mathematics and *g*), providing evidence in support of the Generalist Genes hypothesis. Bivariate heritabilities (Table 5) are about 50%, indicating that about half of the phenotypic correlations across *g*,reading and mathematics are mediated genetically. The Cholesky analysis (Table 6 and left side of Fig. 2) also indicates that, independent of *g*, there is no residual genetic overlap between reading and mathematics and that there are significant genetic influences specific to reading and mathematics.

**Table 6:** Reading, mathematics and *g*: multivariate analysis fitting a Cholesky model. Standardized, squared path coefficients for *g*, reading and mathematics

Measure	A_1_	A_2_	A_3_
*g*	0.44 (0.36–0.53)		
Reading	0.14 (0.08–0.22)	0.24 (0.15–0.32)	
Mathematics	0.27 (0.19–0.37)	0.01(0.00–0.05)	0.20 (0.12–0.27)

	C_1_	C_2_	C_3_
*g*	0.27 (0.19–0.35)		
Reading	0.20 (0.12–0.30)	0.05 (0.00–0.11)	
Mathematics	0.18 (0.11–0.27)	0.03 (0.00–0.09)	0.02 (0.00–0.09)

	E_1_	E_2_	E_3_
*g*	0.28 (0.26–0.31)		
Reading	0.01 (0.01–0.02)	0.35 (0.32–0.39)	
Mathematics	0.01 (0.01–0.02)	0.00 (0.00–0.01)	0.26 (0.24–0.30)

Likelihood ratio χ^2^ test with Δdf compared with saturated phenotypic model: 22.8, 24df, *P* = 0.53. Sample-size-adjusted Bayesian Information Criterion = −17658.

95% confidence intervals in parentheses.

A, additive genetic influence; C, shared environmental influence; E, non-shared environmental influence.

**Table 5:** Reading, mathematics and *g*: multivariate analysis fitting a correlated factors model. Genetic and environmental correlations, bivariate heritability and environmental influence (proportion of phenotypic correlation mediated by A, C and E)

	Reading and mathematics	Reading and *g*	Mathematics and *g*
Correlation			
*r_A_*	0.57 (0.45–0.71)	0.61 (0.48–0.75)	0.75 (0.65–0.86)
*r_C_*	0.94 (0.75–1.00)	0.89 (0.72–1.00)	0.89 (0.73–1.00)
*r_E_*	0.15 (0.08–0.21)	0.19 (0.12–0.25)	0.22 (0.15–0.28)
Mediation of *r_P_*			
A (*a_x_a_y_r_A_/r_P_*)	0.47 (0.35–0.61)	0.46 (0.34–0.59)	0.55 (0.45–0.66)
C (*c_x_c_y_r_C_/r_P_*)	0.44 (0.32–0.54)	0.43 (0.32–0.53)	0.35 (0.25–0.44)
E (*e_x_e_y_r_E_/r_P_*)	0.09 (0.05–0.13)	0.11 (0.07–0.15)	0.10 (0.07–0.13)

*r_A_, r_C_, r_E_* = genetic, shared environmental and non-shared environmental correlations. Model fit statistics are reported in the footnote to Table 6.

95% confidence intervals in parentheses.

Shared environment also shows substantial overlap between reading, mathematics and *g*, contributing almost as much as genetics to their phenotypic correlations and yielding correlations of 0.89–0.94. Non-shared environment, which includes error of measurement, is the chief contributor to differences between abilities, accounting for only about 10% of the phenotypic correlations and yielding correlations of 0.15–0.22.

## Discussion

Using direct tests of cognitive ability, reading and mathematics in the largest representative twin sample to date, the present study provides conclusive evidence in favor of the Generalist Genes hypothesis, the first time it has been tested by direct assessment of cognitive abilities in a large sample. The key results are the genetic correlations of 0.57 between reading and mathematics, 0.61 between reading and *g*, and 0.75 between mathematics and *g*, which are similar to the average result for previous multivariate genetic studies (Markowitz *et al.* 2005; Plomin & Kovas 2005). Despite the large sample, 95% confidence intervals range from 0.45 to 0.86 across the three domains, indicating that the differences in genetic correlations are not significant, and only permitting the conclusion that all three genetic correlations are substantial. The genetic correlations between domains imply that genetic correlations within domains would be even higher, and this is what research suggests (Plomin & Kovas 2005). For example, genetic correlations have been reported to be about 0.90 between reading processes such as word recognition, orthographic coding and phonological decoding (Gayan & Olson 2003), about 0.90 between mathematical computation, application and comprehension (Kovas *et al.* 2007), and about 0.80 between verbal and spatial abilities (Petrill 2002). Likewise, the correlations between shared environmental factors are strong in this childhood sample, accounting for about 40% of the phenotypic correlation between traits. We would expect the shared environmental contribution to the etiology of *g* to diminish throughout childhood and into adolescence while the genetic contribution increases (Boomsma 1993). In contrast, the non-shared environment accounts for very little of the phenotypic correlation between traits, contributing largely to discrepancies in cognitive profiles.

The Generalist Genes hypothesis proposes that some, but not all, genetic effects are general – what is novel is the substantial *extent* to which genetic effects are general. Nonetheless, these genetic correlations are not 1.0, suggesting that there are also genetic effects specific to each domain. The Cholesky analysis (Table 6, left side of Fig. 2) indicates significant domain-specific genetic variance for both reading and mathematics.

These results predict that, when genes are identified that account for the substantial heritability of reading, mathematics and *g*, genes associated with one domain such as reading are highly likely to be associated as well with mathematics and *g*. The Generalist Genes hypothesis implies that molecular genetic attempts to identify genes will profit from targeting what is in common among cognitive domains, as well as what is specific to each (Butcher *et al.* 2006).

For neuroscience, the general effect of genes across such different domains warrants consideration of the possibility that these genes will be found to have similarly general effects across brain structures and functions (Kovas & Plomin 2006). For example, the basic synapse comprises 1000 proteins, with 80% of unknown function in the nervous system (Grant 2006; Jordan & Ziff 2006). The most well-understood synaptic system, the neurotransmitter receptor complex *N*-methyl-d-aspartate receptor complex (NRC/MASC), includes 186 proteins that have been implicated in synaptic plasticity and a wide range of cognitive processes (Pocklington *et al.* 2006). Identifying generalist genes on the basis of their association with downstream cognitive processes could facilitate a systems approach to brain organization (Armstrong *et al.* 2006) because the genes are all anchored in these functional cognitive products of the brain.

For example, genome-wide association studies guided by quantitative genetic findings are beginning to identify sets of polymorphisms associated with cognitive abilities (Butcher *et al.* 2005; Meaburn *et al.* 2007), enabling a top-down approach complementary to bottom-up functional genomic analysis: an approach we have termed behavioral genomics (Harlaar *et al.* 2005; Haworth *et al.* 2007). This allows us to take sets of genes related to cognitive abilities and look at them from a variety of perspectives: multivariate (are the genes associated with one cognitive trait also associated with other cognitive traits?), longitudinal (do the associations reflect changes in the heritability of a trait across time?) and environmental (are the genes associated with a cognitive trait also associated with relevant environments or does the environment influence the strength of the association between the genes and the trait?). We anticipate that the patterns of association emerging from these studies will reflect both the genetic and environmental etiology of cognition derived from quantitative genetics, and the biological systems underlying those cognitive processes arrived at through functional genomics. In this sense, generalist genes could integrate top-down and bottom-up approaches to the systems biology of the brain.

## References

[b1] Armstrong JD, Pocklington AJ, Cumiskey MA, Grant SG (2006). Reconstructing protein complexes: from proteomics to systems biology. Proteomics.

[b2] Birnbaum MH (2004). Human research and data collection via the internet. Annu Rev Psychol.

[b3] Boomsma DI, Bouchard TJ, Propping P (1993). Current status and future prospects in twin studies of the development of cognitive abilities: infancy to old age. Twins as a Tool of Behavioral Genetics.

[b4] Boomsma D, Busjahn A, Peltonen L (2002). Classical twin studies and beyond. Nat Rev Genet.

[b5] Butcher LM, Meaburn E, Knight J, Sham P, Schalkwyk LC, Craig IW, Plomin R (2005). SNPs, microarrays, and pooled DNA: identification of four loci associated with mild mental impairment in a sample of 6000 children. Hum Mol Genet.

[b6] Butcher LM, Kennedy JK, Plomin R (2006). Generalist genes and cognitive neuroscience. Curr Opin Neurobiol.

[b7] Eaves LJ, Eysenck H, Martin NG (1989). Genes, Culture, and Personality: An Empirical Approach.

[b8] Eley TC, Everitt BJ, Howell D (2005). Sex-limitation models. Encyclopedia of Statistics in Behavioral Science.

[b9] Fisher SE, Francks C (2006). Genes, cognition and dyslexia: learning to read the genome. Trends Cogn Sci.

[b10] Galsworthy MJ, Dionne G, Dale PS, Plomin R (2000). Sex differences in early verbal and non-verbal cognitive development. Dev Sci.

[b11] Gayan J, Olson RK (2003). Genetic and environmental influences on individual differences in printed word recognition. J Exp Child Psychol.

[b12] Grant SG (2006). The synapse proteome and phosphoproteome: a new paradigm for synapse biology. Biochem Soc Trans.

[b13] Harlaar N, Butcher L, Meaburn E, Sham P, Craig IW, Plomin R (2005). A behavioural genomic analysis of DNA markers associated with general cognitive ability in 7-year-olds. J Child Psychol Psychiatry.

[b14] Haworth CMA, Harlaar N, Kovas Y, Davis OSP, Oliver B, Hayiou-Thomas ME, Frances J, Busfield P, McMillan A, Dale PS, Plomin R (2007). Internet cognitive testing of large samples needed in genetic research. Twin Res Hum Genet.

[b15] Haworth CMA, Meaburn EL, Harlaar N, Plomin R (2007). Reading and generalist genes. Mind Brain Educ.

[b16] Jordan BA, Ziff EB (2006). Getting to synaptic complexes through systems biology. Genome Biol.

[b17] Kovas Y, Plomin R (2006). Generalist genes: implications for the cognitive sciences. Trends Cogn Sci.

[b18] Kovas Y, Petrill SA, Plomin R (2007). The origins of diverse domains of mathematics: generalist genes but specialist environments. J Educ Psychol.

[b19] Markowitz EM, Willemsen G, Trumbetta SL, Beijsterveldt CEM, Boomsma DI (2005). The etiology of mathematical and reading (dis)ability covariation in a sample of Dutch twins. Twin Res Hum Genet.

[b20] Markwardt FC (1997). Peabody Individual Achievement Test-Revised (Normative Update) Manual.

[b21] Martin N, Boomsma DI, Machin G (1997). A twin-pronged attack on complex traits. Nat Genet.

[b22] Meaburn EL, Harlaar N, Craig IW, Schalkwyk LC, Plomin R (2007). QTL association scan of early reading disability and ability using pooled DNA and 100K SNP microarrays in a sample of 5,500 children. Mol Psychiatry.

[b23] Neale MC, Boker SM, Xie G, Maes HH (2006). Mx: Statistical Modeling.

[b24] nferNelson (2001). Maths 5-14 Series.

[b25] Oliver BR, Plomin R (2007). Twins Early Development Study (TEDS): a multivariate, longitudinal genetic investigation of language, cognition and behavior problems from childhood through adolescence. Twin Res Hum Genet.

[b26] Petrill SA, Sternberg RJ, Grigorenko EL (2002). The case for general intelligence: a behavioral genetic perspective. The General Factor of Intelligence: How General Is It?.

[b27] Plomin R, DeFries JC (1979). Multivariate behavioral genetic analysis of twin data on scholastic abilities. Behav Genet.

[b28] Plomin R, Kovas Y (2005). Generalist genes and learning disabilities. Psychol Bull.

[b29] Plomin R, DeFries JC, McClearn GE, McGuffin P Behavioral Genetics.

[b30] Pocklington AJ, Cumiskey M, Armstrong JD, Grant SG (2006). The proteomes of neurotransmitter receptor complexes form modular networks with distributed functionality underlying plasticity and behaviour. Mol Syst Biol.

[b31] Raven JC, Court JH, Raven J (1996). Manual for Raven's Progressive Matrices and Vocabulary Scales.

[b32] Rijsdijk FV, Sham PC (2002). Analytic approaches to twin data using structural equation models. Brief Bioinform.

[b33] Shrout PE, Fleiss J (1979). Intraclass correlations: uses in assessing rater reliability. Psychol Bull.

[b34] Trouton A, Spinath FM, Plomin R (2002). Twins Early Development Study (TEDS): a multivariate, longitudinal genetic investigation of language, cognition and behaviour problems in childhood. Twin Res.

[b35] Wechsler D (1992). Wechsler Intelligence Scale for Children – Third Edition UK (WISC-IIIUK) Manual.

